# Public Attitudes About COVID-19 in Response to President Trump's Social Media Posts

**DOI:** 10.1001/jamanetworkopen.2021.0101

**Published:** 2021-02-01

**Authors:** Dominic Arjuna Ugarte, William G. Cumberland, Lidia Flores, Sean D. Young

**Affiliations:** 1 Department of Emergency Medicine, School of Medicine, University of California, Irvine; 2 Department of Biostatistics, University of California, Los Angeles; 3 Department of Informatics, School of Information and Computer Sciences, University of California, Irvine

## Abstract

This cross-sectional study examines public beliefs about the coronavirus disease 2019 (COVID-19) pandemic in response to President Trump’s social media posts during and after his infection with the virus.

## Introduction

Misinformation has hampered health officials’ ability to control the coronavirus disease 2019 (COVID-19) pandemic.^1,2,3^ Public beliefs about COVID-19 are influenced by political ideology and media.^4^ In particular, President Trump’s tweets regarding his clinical status may have affected the beliefs of US individuals about the pandemic’s severity. Surveys and interviews, typically used to gauge public perspectives, have collection lag times.^5^ Accordingly, we sought to use near real-time social media data to capture the public’s changing COVID-19–related attitudes during President Trump’s recent infection.

## Methods

For this cross-sectional study, from September 23 to October 8, 2020, we collected 22 800 tweets regarding public views on COVID-19 using Twitter’s free application programming interface. Search criteria included COVID-19–related keywords (eg, *COVID-19*, *coronavirus*) within 15 characters of a supporting (eg, *serious*, *real*) or contradicting (eg, *hoax*, *#CovidHoax*) adjective or hashtag. Tweets were collected from “swing states” (Florida, Michigan, Colorado, Iowa, Minnesota, Nevada, New Hampshire, North Carolina, Ohio, Pennsylvania, Virginia, and Wisconsin) (Box), were restricted to individual accounts, excluded retweets, and were obtained from 1 of 3 periods: (1) September 23 to October 1, 2020 (before President Trump’s tweet that he had contracted COVID-19); (2) October 1 to October 5, 2020 (between his infection announcement and his tweet, “Don’t be afraid of COVID”); and (3) October 5 to October 8, 2020 (after his “Don’t be afraid” tweet). This study and the consent process were exempted by the University of California, Irvine, institutional review board because all data were public. The study followed the Strengthening the Reporting of Observational Studies in Epidemiology (STROBE) reporting guideline.

Box. **Example Tweets by Belief in the Severity of COVID-19^a^**

**COVID-19 Is a Hoax**

“Covid is fake”
“Pandemic = myth. #CoronaHoax”

**COVID-19 Is Real But Not Serious**

“It's time to get real about Covid. Getting it is far from a death sentence. In fact the survival rate is greater than 98%. We should be vigilant but if we get it we get it. We don't like the flu but we manage. Time to go back to life as we knew it before this hype”
“cdc has said most of the mask people wear are not even effective there has been no social distancing during riots no one seems to care. Many of those 210k people didn't even really die from the actual Chinese virus just go back to getting false info from fake news”

**COVID-19 Is Real and Serious**

“Stay home...even if you don’t care or believe this entire Pandemic is a hoax! It’s time to CHANGE your behavior #Covid19isarealthing”
“Wow, a little covid and Twitter goes silent.....come on man, it's all a hoax right? #GotWhatYouDeserved”

**Unrelated or No Stance**

“Like i said to my mom last night. how come real tax payers are paying thousands in medical bills for covid. why isn’t trump paying for his medical bills?”
“Not as fake as Trump claiming to have Covid #TrumpCovidHoax #TrumpNeverHadCovid19”

Abbreviation: COVID-19, coronavirus disease 2019.

^a^Taken from 5945 tweets identified from September 23, 2020, to October 8, 2020. Tweets were restricted to “swing states” (Florida, Michigan, Colorado, Iowa, Minnesota, Nevada, New Hampshire, North Carolina, Ohio, Pennsylvania, Virginia, and Wisconsin), individual accounts, and non-retweets.

We randomly selected and manually labeled 3000 tweets from each period into 1 of 4 categories: (1) COVID-19 is not real, (2) COVID-19 is real but not serious, (3) COVID-19 is real and serious, and (4) unrelated or no stance (Box). Interrater reliability among 4 labelers on 93 randomly selected tweets yielded a Krippendorff α of 0.47. Tweets were restricted to 1 tweet per Twitter username by discarding any tweets after the first. Unrelated tweets from each period (301, 354, and 788 tweets, respectively) were removed, leaving 5945 tweets for analysis. A χ^2^ test was used to assess differences in tweet proportions for each category across periods. Significance was set at a 2-sided *P* = .05. Analyses were performed using Excel, version 1808 (Microsoft Corp).

## Results

We found statistically significant differences in the proportion of COVID-19 severity categories between periods 1 and 2 (χ^2^ = 569.9; *P* < .001), 2 and 3 (χ^2^ = 283.5; *P* < .001), and overall (χ^2^ = 582.9; *P* < .001) (with Bonferroni correction) (Table). Most tweets showed that COVID-19 was perceived as a real and serious issue across all periods (period 1, 1441 [67.3%]; period 2, 2060 [95.6%]; period 3, 1284 [78.0%]). Initially, 417 tweets [19.5%] showed the belief that COVID-19 was a hoax, and 284 (13.3%) showed the belief that it was not serious. After President Trump tweeted about his infection, 66 tweets (3.1%) showed the belief that COVID-19 was a hoax, and 30 (1.4%) showed the belief that it was not serious. This pattern reversed slightly after he tweeted, “Don’t be afraid of COVID-19” (hoax, 164 [10.0%]; not serious, 199 [12.0%]).

**Table.** COVID-19–Related Beliefs Over Time^a^

COVID-19 severity category	Tweets, No. (%) [95% CI]		
	Period 1 (n = 3800)	Period 2 (n = 13 800)	Period 3 (n = 5200)
Hoax	417 (19.5) [17.8-21.1]	66 (3.1) [2.3-3.8]	164 (10.0) [8.5-11.4]
Not serious	284 (13.3) [11.8-14.7]	30 (1.4) [0.9-1.9]	199 (12.0) [10.5-13.7]
Serious	1441 (67.3) [65.3-69.3]	2060 (95.6) [94.7-96.4]	1284 (78.0) [76.0-80.0]
Total	2142 (100)	2156 (100)	1647 (100)

Abbreviation: COVID-19, coronavirus disease 2019.

^a^ Period 1 was from September 23 to October 1, 2020 (before President Trump’s tweet that he had contracted COVID-19); period 2 was from October 1 to October 5, 2020 (between his infection announcement and his tweet, “Don’t be afraid of COVID”); and period 3 was from October 5 to October 8, 2020 (after his “Don’t be afraid” tweet).

## Discussion

These results suggest that some members of the public adapted beliefs in response to President Trump’s tweets and that social media may be used as a near real-time data source to capture these changing perspectives, including COVID-19–related misinformation. This analysis has several immediate public health implications. First, health departments, political leaders, and influencers should leverage social media to support science on health issues, especially during pandemics.^3^ Second, social media can be used to assess the public’s changing responses to public health–related communications. Third, social media analysis can and should be explored to identify sources of and ways to combat misinformation.

Limitations of this study include Twitter not being representative of the general population.^6^ Although we manually reviewed tweets, restricted data to individual accounts, excluded retweets, and limited tweets per Twitter username, online manipulation (eg, “bots”) remains a potential issue. In addition, we did not have demographic data, such as political affiliation, limiting more detailed analyses.^6^ Overall, the results suggest that social media can be used as a tool to capture public perspectives, identify misinformation, and aid public health efforts.
